# Risk factors for shoulder re-dislocation after arthroscopic Bankart repair

**DOI:** 10.1186/s13018-014-0053-z

**Published:** 2014-07-04

**Authors:** Hideaki Shibata, Masafumi Gotoh, Yasuhiro Mitsui, Yoshihiro Kai, Hidehiro Nakamura, Tomonoshin Kanazawa, Takahiro Okawa, Fujio Higuchi, Masahiro Shirahama, Naoto Shiba

**Affiliations:** 1 Department of Orthopaedic Surgery, Kurume University, 67 Asahi-machi, Kurume 830-0011, Fukuoka, Japan; 2 Department of Orthopaedic Surgery, Kurume University Medical Center, 155-1 Kokubu-machi, Kurume 839-0863, Fukuoka, Japan; 3 Department of Rehabilitation, Kyoto Tachibana University, Kyoto 607-8175, Japan

**Keywords:** Risk factor, Arthroscopic Bankart repair, Re-dislocation

## Abstract

**Background:**

Recent studies have shown effective clinical results after arthroscopic Bankart repair (ABR) but have shown several risk factors for re-dislocation after surgery. We evaluated whether patients are at a risk for re-dislocation during the first year after ABR, examined the recurrence rate after ABR, and sought to identify new risk factors.

**Methods:**

We performed ABR using bioabsorbable suture anchors in 102 consecutive shoulders (100 patients) with traumatic anterior shoulder instability. Average patient age and follow-up period was 25.7 (range, 14–40) years and 67.5 (range, 24.5–120) months, respectively. We evaluated re-dislocation after ABR using patient telephone interviews (follow-up rate, 100%) and correlated re-dislocation with several risk factors.

**Results:**

Re-dislocation after ABR occurred in nine shoulders (8.8%), of which seven sustained re-injuries within the first year with the arm elevated at 90° and externally rotated at 90°. Of the remaining 93 shoulders without re-dislocation, 8 had re-injury under the same conditions within the first year. Thus, re-injury within the first year was a risk for re-dislocation after ABR (*P* < 0.001, chi-squared test). Using multivariate analysis, large Hill-Sachs lesions (odds ratio, 6.77, 95% CI, 1.24–53.6) and <4 suture anchors (odds ratio, 9.86, 95% CI, 2.00–76.4) were significant risk factors for re-dislocation after ABR.

**Conclusions:**

The recurrence rate after ABR is not associated with the time elapsed and that repair strategies should augment the large humeral bone defect and use >3 anchors during ABR.

## Background

Arthroscopic Bankart repair (ABR) provides acceptable results for recurrent anterior shoulder instability. However, recent studies have shown recurrent rates of 4%–19% [[1]–[5]]. Several factors, including a young age at the time of surgery, male sex, shoulder instability on both sides, joint hyperlaxity, participation in collision sports, early return to contact sports, the size of the humeral defect (Hill-Sachs lesion), and bone defects have been associated with the recurrent instability [[6]–[10]]. In addition, a recent study showed that 55% of the re-dislocations after ABR occurred within the first year, and thereafter, the recurrence rate decreased for up to 5 years [[11]]. Therefore, these results prompted us to evaluate whether the patients with primary ABR are at risk for re-dislocation during the first year after the surgery. In addition, the present study examined the recurrence rate after ABR and sought to identify new risk factors.

## Methods

### Patients

We treated 102 consecutive shoulders (100 patients) using ABR for traumatic anterior shoulder instability from February 2002 to December 2010 at our institute. The average patient age and follow-up period was 25.7 (range, 14–40) years and 67.5 (range, 24.5–120) months, respectively. Inclusion criteria included: (1) recurrent anterior shoulder instability after an apparent traumatic episode, (2) at least three dislocation/subluxations before the surgery, (3) a Bankart lesion or anterior labral periosteal sleeve avulsion lesion confirmed during arthroscopy, (4) an arthroscopic capsulolabral repair achieved using three or more suture anchors, and (5) a minimum of 2 years follow-up was completed by telephone interview. Exclusion criteria included: (1) multidirectional instability, (2) revision Bankart repairs, and (3) full-thickness rotator cuff tears.

Preoperative radiographic imaging, consisting of anteroposterior, scapular Y, and axillary views, was obtained to evaluate the glenoid shape of the glenoid and the presence of any bony (i.e., Bankart or Hill-Sachs) lesions. Contrast magnetic resonance imaging of the affected shoulder was evaluated for the presence of a Bankart lesion and any other shoulder injury before surgery.

Institutional review board approval by the Kurume University Ethic Committee and verbal consent at the time of phone interview were obtained.

### Surgical procedure

A single surgeon (MG) performed all the ABR procedures, using the same procedure, with the patients under general anesthesia in a beach chair position. A standard posterior portal was created and used as a viewing portal. Subsequently, the glenohumeral joint was inspected and pathology verified. Anterior and anterosuperior portals were established above the subscapularis tendon and anterolateral to the acromion, respectively. After mobilization of the inferior glenohumeral ligament complex from the glenoid neck up to a 7 to 5 o'clock position, the glenoid neck and its articular edge were decorticated with a motorized shaver and ring curette to facilitate healing of the repaired capsulolabrum.

The first anchor was placed at the 5 o'clock position (right shoulder), using a biodegradable push-in suture anchor (Panalock, 2002–2005 and Panalock loop, approximately 2005, DePuy Mitek, Inc., Raynham, MA, USA) with an eyelet single loaded with a no. 2 non-absorbable suture (2002–2010, Ethibond, DePuy Mitek, Inc.; 2010–2012: FiberWire, Arthlex, Inc., Naples, FL, USA). To pass the suture through the anterior portal, a shuttle relay was passed through the labrum and brought out through the anterosuperior portal. The capsulolabrum was shifted as superior to the glenoid as possible. A standard sliding knot was tied on the capsulolabral side. These steps were repeated for each anchor used in the repair and capsular shift. Three to six anchors were used for the capsulolabral repair.

The patients were kept in a shoulder sling with an abduction pillow at neutral rotation and 20° abduction postoperatively. Three weeks after the surgery, progressive self-assisted shoulder elevation and external rotation were initiated. Active range-of-motion exercise was permitted at 6 weeks postoperatively, rotator cuff strengthening at 12 weeks postoperatively, and full participation in sports at 6 months postoperatively.

### Outcome measures

We successfully contacted all 100 patients who underwent ABR in our institution via telephone. A re-visit for postoperative evaluation was requested although most of the patients declined the visit. Therefore, the patients' present status, including postoperative injury and re-dislocation with either subluxation or complete dislocation, was inquired via phone. The mean follow-up time of the phone survey was at 67.5 months (range, 24.5–120 months).

After collecting the patient's information, correlations of several risk factors were determined, including gender, injured side, age at first dislocation and surgery, arm dominance, type of sport (collision, contact, overhead, or others), waiting time prior to surgery, number of dislocations preoperatively, number of suture anchors used, superior labrum anterior and posterior (SLAP) lesion, and tear of the capsular. The patient demographic data are shown in Table 1.

**Table 1:** Patient demographic data

	**Range**	**Mean ± SD**	**Median**	**Number (*n*)**	**Percentage (%)**
Age (y)	16–42	25.7 ± 9.66	23.5		
Age at first dislocation (y)	12–40	22.4 ± 8.6	20.0		
Gender					
Male				81	79
Female				21	21
Injury to dominant arm					
No				42	41
Yes				60	59
Injured side					
Right				58	57
Left				44	43
Dominant side					
Right				91	89
Left				11	11
Type of sport					
No sport				42	41
Collision				21	20
Contact				14	14
Overhead				25	25
Waiting time to surgery (months)					
>6				70	69
<6				32	31
Number of re-dislocations prior to surgery					
<5				32	31
>5				70	69
Number of suture anchor used					
3				47	46
4				41	40
5				14	14
SLAP lesion					
Yes				1	29
No				8	64
Tear of capsular					
No				86	85
Yes				16	15

*SLAP* superior labrum anterior and posterior.

### Evaluation of bony defect

Since previous studies have suggested that both glenoidal and humeral head bone defects are closely associated with re-dislocation after ABR [[6],[12],[13]], these bony defects were measured using an arthroscopic probe technique [[5]]. For glenoid bone defects, using the anterosuperior-viewing portal, a probe with 3-mm calibrated marks was placed through the posterior portal across the glenoid so that its tip rested on the bare spot. The distance from the center of the bare spot to the posterior glenoid rim was then measured. The probe was then used to measure the distance from the anterior glenoid rim to the center of the bare spot. Finally, the probe was used to measure the distance from the center of the bare spot to the inferior glenoid rim [[14],[15]]. Humeral head defects (Hill-Sachs lesions) were also measured with arthroscopic probe techniques, based on an estimation of the width, depth, and length, as measured intraoperatively with the arthroscopic probe [[5]]. The critical size of a Hill-Sachs lesion that causes instability is thought to be a volume > 250 mm^3^ [[16]–[18]]; thus, such lesions described ‘large Hill-Sachs lesions’.

### Statistical analysis

The software JMP (SAS Institute Inc., Cary, NC, USA) was used for statistical analysis. Since the development of re-dislocation was a time-dependent outcome variable, we used survival methodology (Kaplan-Meier curve) to examine the probability of re-dislocation occurring after ABR, by setting re-dislocation as the end-point. Student's *t* test or chi-squared test was used to compare the bony defect size between the patients with or without re-dislocation. A chi-squared test was used to examine the correlations between the clinical parameters and re-dislocation after ABR. Logistic multivariate analysis was then performed to further evaluate the significant parameters obtained from the Pearson's chi-squared test, accompanied by the odds ratio with 95% confidence intervals. The data are expressed as the mean values with the standard deviation. A *P* value < 0.05 was considered significant.

## Results

### Postoperative re-dislocation

**Figure 1:**
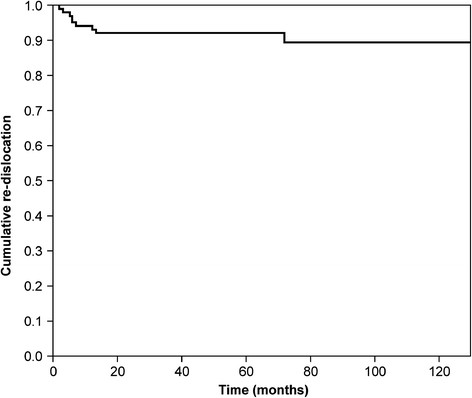
Kaplan-Meier curve of the re-dislocation rate over time.

Of the 102 shoulders treated with ABR, a total of 9 (8.8%) experienced re-dislocation (Figure 1). Of these, seven shoulders were re-injured within the first year with the arm elevated at 90° and externally rotated at 90°. Another experienced re-injury and re-dislocation at 15 months and 6 years after surgery. Thus, most re-dislocations (78%) occurred within the first year after ABR. Of the nine patients who had a re-dislocation, two patients underwent re-operation, and the remaining seven patients were treated non-operatively or refused operation. Of the 93 shoulders without re-dislocation, 7 shoulders had a traumatic injury within the first year under the same conditions (90° elevation and 90° external rotation). The shoulders were re-dislocated during contact and overhead sports (*n* = 2), as well as activities of daily livings (*n* = 5). Thus, re-injury within the first year proved to be a risk for re-dislocation after ABR (*P* < 0.001, chi-squared test, Table 2).

**Table 2:** Correlation between injury within the first year after surgery and postoperative re-dislocation

**Injury within 1 year**	**Re-dislocation (+)**	**Re-dislocation (−)**	**Total**
Yes	7*	8	15
No	2	85	87
Total	9	93	102

*Statistical significance.

### Bony defects

Seventy-one of the 102 shoulders (69.6%) had a Hill-Sachs lesion and 37 (37%) had a large defect of the humeral head (>250 mm^3^) [[14]], which occurred at a significantly greater frequency in shoulders with re-dislocation than in those without re-dislocation (7 of 9 shoulders (78%) vs. 30 of 93 shoulders (32%), *P* < 0.001, chi-squared test). Significantly larger defect were also seen in the shoulders with re-dislocation compared with those without re-dislocation (834 ± 485 mm^3^ vs. 190 ± 255 mm^3^, *P* < 0.01, Student's *t* test) (Table 3).

**Table 3:** Comparison of bony defects between patients with or without re-dislocation after surgery

	**Re-dislocation (+)**	**Re-dislocation (−)**	***P*** **value**
Hill-Sachs lesion (mm^3^)	834 ± 485	190 ± 255	<0.01
Glenoid defect (%)	29.5 ± 3.3	19.6 ± 1.9	<0.01

Mean **±** SD.

A glenoid defect was noted in 20 of the 102 shoulders (19%) and was more prominent in the re-dislocated compared to the non-re-dislocated shoulders (4 of 9 shoulders (44%) vs. 16 of 93 shoulders (17%), *P* = 0.049, chi-squared test). A critical defect >20% [[18]] was noted in three dislocated and seven non-dislocated shoulders (9.8%).

### Risk factors for re-dislocation after ABR

Using a chi-squared test, we found that a large Hill-Sachs lesion (>250 mm^3^) [[5]] (*P* = 0.013), glenoid bone defect (>20%), and less than four suture anchors (*P* = 0.011) were significant risk factors for recurrence after ABR (Table 4). In contrast, there was no evidence of a relationship between re-dislocation and other factors such as age at the time of first dislocation (*P* = 0.27), gender (*P* = 0.68), the number of previous dislocations before ABR (*P* = 0.28), waiting time prior to surgery (*P* = 0.30), arm dominance (*P* = 0.59), injured side (*P* = 0.49), SLAP lesion (*P* = 0.27), or capsular tear (*P* = 0.62).

**Table 4:** Analysis of risk factors for re-dislocation after ABR by a chi-squared test

**Variable**	***P*** **value**
Large Hill-Sachs lesions	0.010*
Number of anchors	0.010*
Glenoid bone loss (>20%)	0.042*

*Significance at *P* < 0.05.

When the variables that demonstrated significance with the chi-squared test were further entered into multivariate analysis, the number of suture anchors used (odds ratio, 9.56; 95% CI, 1.99-71.4) and large Hill-Sachs lesions (odds ratio, 9.14; 95% CI, 1.90-68.3) remained independently predictive (Table 5).

**Table 5:** Analysis of risk factors for re-dislocation after ABR by multivariate analysis

**Variable**	**P value**	**Odds ratio**	**95% CI**
Large Hill-Sachs lesions	0.026*	6.77	1.24–53.6
Number of anchors	0.0041*	9.86	2.00–76.4
Glenoid bone loss	0.148		

*Significance at *P* < 0.05.

### Complications

No complications related to the anchors or sutures were noted in the present series, although one patient had an acute superficial infection that was readily resolved with antibiotics administration.

## Discussion

The present study successfully evaluated shoulder re-dislocation after ABR with 100% follow-up through phone survey. We found a significant association between re-dislocation and preoperative risk factors, including a large Hill-Sachs lesion and the use of less than four suture anchors. Re-dislocation primarily and significantly occurred within the first year after the operation. Thus, we confirmed that the risk of re-dislocation after ABR is greater in the first year compared to subsequent postoperative years, indicating that the recurrence rate after ABR is not associated with the time elapsed and suggests the importance of extra care within this period.

Ahmed et al. [[11]] have also shown that patients were at risk for re-dislocation within the first year after ABR, and thereafter, the rate of recurrence decreased. Considering that most patients with a high-predicted risk of re-dislocation do not develop recurrent instability, while others with a few risk factors may experience failure after ABR [[11]], the existence of other factors (e.g., compliance with postoperative immobilization, re-injury after ABR, increase of general activity, and genetic predisposition) [[11]] may be related to the intensive occurrence of re-dislocation within the first year after ABR. Alternatively, the repaired capsulolabral complex may not have healed during the first year since the healing process of the repaired site has not yet been completely elucidated.

Long-term follow-up studies for ABR have indicated that recurrence rates increase with time [[9],[19]]. Castagna et al. [[19]] reported that in 31 of 43 shoulders with ABR, 7 were dislocated (22%) at a mean follow-up of 10.9 years, with 3 of the 7 recurrences developing after 6 years [[19]]. van der Linde et al. [[9]] showed that in 68 of 70 shoulders with ABR, a total of 24 experienced re-dislocation after surgery (35%), with a mean follow-up period of 8–10 years. Re-dislocation occurred in 10 shoulders (15%) within the first 2 years, 7 shoulders (10%) at 2–5 years postoperatively, and 7 shoulders (10%) after 5 years [[9]]. In these studies, two or three suture anchors were used in most cases. As demonstrated in the present study, the use of less than four suture anchors was closely associated with the recurrent instability after ABR [[20]]. Taken together, the use of fewer anchors in long-term studies may explain why the incidence of re-dislocation increased over time.

In line with the report of Ahmed et al. [[11]], the present study demonstrates that the recurrence rate after ABR is not associated with the time elapsed. This previous study used three or more suture anchors in the modern ABR technique with capsular plication [[11]]. This new technique has shown to decrease the recurrence rate to 4%–19% [[1]–[5]]. Our current findings indicate that modern ABR (with capsular plication), using more than three anchors, may further decrease the recurrence rate and prevent re-dislocation within the first year. In a systemic review, patients with two anchors had a 35% recurrence of instability, those with three anchors demonstrated 20%, and those with four or more anchors showed 10% recurrence stability [[8],[21],[22]]. As mentioned previously, the present study consistently found that the use of less than four anchors was a significant risk factor for re-dislocation after ABR.

Previous studies have indicated that a large/engaging Hill-Sachs lesion is significantly involved in re-dislocation after ABR [[23],[24]]. Traumatic anterior shoulder instability is often associated with bone loss from the glenoid, humerus, or both. Bony defects of the glenoid are reported in 5%–56% cases of traumatic anterior shoulder instability [[25]–[28]]. The articular arc deficit of the humeral head allows engagement of the bone defect on the anterior glenoid rim, the so-called engaging Hill-Sachs lesion [[6]]. Enlargement of the bone defect of this lesion is well correlated with the engagement of the glenoid rim [[24],[25],[29],[30]]. Although engagement between the humeral and glenoidal defects were not evaluated in detail in the current study, a large Hill-Sachs lesion was significantly associated with the recurrence after ABR, in line with the results of previous studies [[31]].

Bone loss of >20%–30% is associated with a significant increase in re-dislocation after surgery [[12],[32],[33]]. In the present study, 10% of the patients had a glenoid bone defect greater than 20%, thus affecting the data analysis in this series. A larger sample of patients with ABR may have elicited a significant association with glenoid bone defects and re-dislocation.

The present study has several limitations. First, this study failed to perform direct physical or radiographical examination in all patients who underwent ABR. The patients were phone-interviewed and asked about trauma and/or re-dislocation, and long duration of post-surgery, suggesting the possibility for recall bias, which cannot be ruled out. However, we focused on examining the relationship between clinical parameters and re-dislocation after ABR and successfully contacted all patients by telephone for obtaining details about trauma/re-dislocation after surgery. In addition, the present series was a retrospective, not a prospective study. However, we were able to examine the re-dislocation rate after ABR in all patients, with a mean follow-up of approximately 6 years (range, 2–10 years).

## Conclusions

The present study indicates that a large Hill-Sachs lesion and the number of the suture anchors are significant risk factors for re-dislocation after ABR. Recurrence rate after ABR is not associated with the time elapsed.

## Competing interests

The authors declare that they have no competing interests.

## Authors’ contributions

The design of the study and preparation of the manuscript were done by HS, MG, YM TK, HN, and YK. MS, TO, FH, and NS assisted in the manuscript preparation. All authors read and approved the final manuscript.
